# Effect of acupuncture with donepezil based on syndrome differentiation on cognitive function in patients with mild-to-moderate Alzheimer’s disease: a study protocol for a multicenter randomized controlled trial

**DOI:** 10.1186/s13063-022-06532-1

**Published:** 2022-08-19

**Authors:** Qin-Hui Fu, Jian Pei, Hou-guang Zhou, Tao Wang, Yi-jun Zhan, Lin Tao, Jia Xu, Qian Zhou, Liao-yao Wang

**Affiliations:** 1 Acupuncture Department, Longhua Hospital, Shanghai University of Traditional Chinese Medicine, 725 South Wanping Road, Shanghai, 200032 China; 2 Department of Geriatrics, Huashan Hospital, Fudan University, Shanghai, 200040 China; 3 Alzheimer’s Disease and Related Disorders Center, Shanghai Mental Health Center, School of Medicine, Shanghai Jiaotong University, Shanghai, 200030 China

**Keywords:** Alzheimer’s disease, Acupuncture, Syndrome differentiation, Randomized controlled trial, Study protocol

## Abstract

**Background:**

There has been a rapid increase in the worldwide prevalence of Alzheimer’s disease (AD). Previous studies have shown that acupuncture can improve neurological and cognitive function; however, the utility of applying acupuncture in patients with AD remains unclear. This study protocol describes a clinical trial for evaluating the efficacy and safety of acupuncture based on syndrome differentiation with donepezil hydrochloride on cognitive function in patients with AD.

**Methods/design:**

This multicenter randomized controlled trial commenced on February 1, 2019, at the Shanghai Longhua Hospital of TCM, Shanghai Huashan Hospital of Fudan University, and Shanghai Mental Health Center, and will conclude on June 30, 2022. The study will recruit 184 patients randomly divided into an acupuncture group or a control group at a 1:1 ratio. All participants will receive donepezil hydrochloride (5 mg/day), and those in the acupuncture group will receive acupuncture based on syndrome differentiation with donepezil for 12 weeks. The primary outcome will be the post-treatment change in the Alzheimer’s Disease Assessment Scale-cognition score at 12 weeks. The secondary outcomes will be the efficacy scores of the Minimum Mental State Examination, Alzheimer’s Disease Cooperative Research Activity-Daily Life, and Quality of Life-Alzheimer’s Disease. All assessments will be performed at baseline, after treatment (week 12), and at follow-up (weeks 24 and 36).

**Discussion:**

This trial may provide high-quality evidence for the efficacy of acupuncture in the treatment of AD. The results of this study will be published in peer-reviewed journals.

**Trial registration:**

ClinicalTrials.govNCT03810794. Registered on 17 January 2019.

## Introduction

### Background

The incidence of Alzheimer’s disease (AD) is increasing worldwide [1], and the aging of the population in particular will accelerate this trend. According to statistics, there are about 7 million people with AD [2], which is the eighth leading cause of death, causing a heavy burden on families and society [3]. In China, the treatment burden of AD has reached $167.74 billion in 2015, accounting for 1.47% of the gross domestic product, and it is expected to reach $2.11 trillion worldwide in 2030 [4]. Unfortunately, it is estimated that by 2050, the number of population over 65 in China will reach 400 million [5]. If calculated based on the prevalence rate of 8.2/1000 person-years over 65 [6], the prevention and treatment of AD will undoubtedly bring a heavy burden to China by 2050.

AD mainly manifests as hippocampal amnesia syndrome. It is characterized by episodic memory impairment, coupled with changes in behavior and emotions as well as a significant reduction in patients’ daily life capability, social interaction, and work ability [7]. The pathophysiological mechanism of AD is complex and its pathogenesis has not yet been elucidated. Typical pathological features of AD include the deposition of extracellular β-amyloid (Aβ) to form senile plaques (SP), neurofibrillary tangles (NFT) composed of intracellular hyperphosphated tau protein, and synapse loss [8, 9]. The most effective treatment for AD involves promoting cholinergic neurotransmission and reducing acetylcholine hydrolysis in the brain [10–13]. Currently, the US Food and Drug Administration has only approved five medicines for AD. Four of the drugs are cholinesterase (AchE) inhibitors, while the fifth, memantine [14], is an inhibitor of the N-methyl-D-aspartic acid receptor (NMDA). A previous meta-analysis reported that taking one of the AchE inhibitors (donepezil hydrochloride) for 24–26 weeks reduced the Alzheimer’s Disease Assessment Scale-Cognitive Subscale (ADAS-cog) score of patients with mild-to-moderate AD by an average of 2.67 points [15]. However, considerable adverse effects of the medicines such as muscle cramps, weakness, nausea, vomiting diarrhea, and insomnia were also reported [16, 17].

Our previous study reviews [18] five non-pharmacological prevention strategies for AD and cognitive decline can effectively supplement drug therapies. Currently, non-drug treatments mainly include exercise intervention, cognitive intervention, music therapy, repetitive transcranial magnetic stimulation, and acupuncture therapy [19]. Among them, acupuncture therapy is a unique non-pharmacologic therapy which can improve the cognitive function of patients [20] through various mechanisms, such as protecting neurons, enhancing neurotransmission, reducing oxidative stress, and reducing Aβ protein deposition [21]. Studies indicate that acupuncture can regulate brain networks by increasing connectively between cognition-related regions, such as the insula, dorsolateral prefrontal cortex, hippocampus, thalamus, inferior parietal lobule, and anterior cingulate cortex, thereby improving cognitive function [22] and acupressure training improved cognitive function of old adults [23].

Recently, several clinical trials have shown that acupuncture can have a positive effect on patients with AD, including improving cognition [24–27]and memory [27, 28], as well as reducing the side effects of medications [29].

Preliminary achievement in experimental studies has been conducted on the mechanism of acupuncture treatment for AD. In amyloid precursor protein/presenilin-1(APP/PS1) transgenic mice, acupuncture reduced the deposition of β-amyloid precursor protein lyase 1 (BACE1), a key protein involved in the production of Aβ peptide; regulated the level of protein kinase A (PKA) and its related substrates (such as long-term potentiation [LTP]) [30]; upregulated the expression of brain-derived neurotrophic factor (BDNF), and promoted neurogenesis [31]. In Senescence-accelerated mouse prone 8 (SAMP8), acupuncture enhanced cell proliferation in dentate gyrus (DG) [32], reduced memory impairment, and increased the level of p-AMPK (phosphor-activated protein kinase) in the hippocampus [33]. Furthermore, in injected A*β1–40* rat model, acupuncture led to improved learning and memory function by suppressing the Notch signaling pathway in the hippocampus [34]. PET studies have shown that acupuncture can increase blood perfusion and metabolism in certain brain regions, including the right amygdala, right hippocampus and thalamus, and brain stem which may have a positive impact on cognition in ibotenic acid injection rats [35, 36].

In acupuncture clinic practice, doctors select varying acupoint formulas based on individual conditions, which are significantly affected by the heterogeneity of patients. Our previous study [37] on the research literature in the past 20 years has implied that heart qi deficiency and kidney essence deficiency are the main TCM syndromes of AD. A randomized controlled trial (RCT) on a formula for tonifying the SHEN (*kidney*) or XIN (*heart*) reported a minimum effective rate of 70.91% (using the Mini-Mental State Examination [MMSE]) after 48 weeks’ treatment in patients with mild-to-moderate AD [38]. However, few studies on acupuncture for AD have employed syndrome differentiation, which could have contributed to inconsistent findings. We previously conducted a retrospective study on 30 patients with mild-to-moderate AD who underwent acupuncture based on kidney or heart differentiation for 3 months in the Acupuncture Department of Longhua Hospital. After the 12-week treatment, the ADAS-cog score decreased by 4.04±4.23 points. These findings encouraged us to further evaluate the role of acupuncture therapy in AD treatment. This clinical trial will aim to evaluate the efficacy and safety of acupuncture based on syndrome differentiation with donepezil on cognitive function, activities of daily living, and quality of life in patients with mild-to-moderate AD. The findings of this clinical trial could contribute toward establishing a normalized program for the treatment of AD with acupuncture.

### Objectives

This study protocol aims to describe a clinical trial for evaluating the efficacy and safety of acupuncture with donepezil hydrochloride on cognitive function in patients with AD and to explore the role of acupuncture based on syndrome differentiation (heart qi deficiency, kidney essence deficiency) in the conventional treatment of AD.

### Trial design

**Fig. 1:**
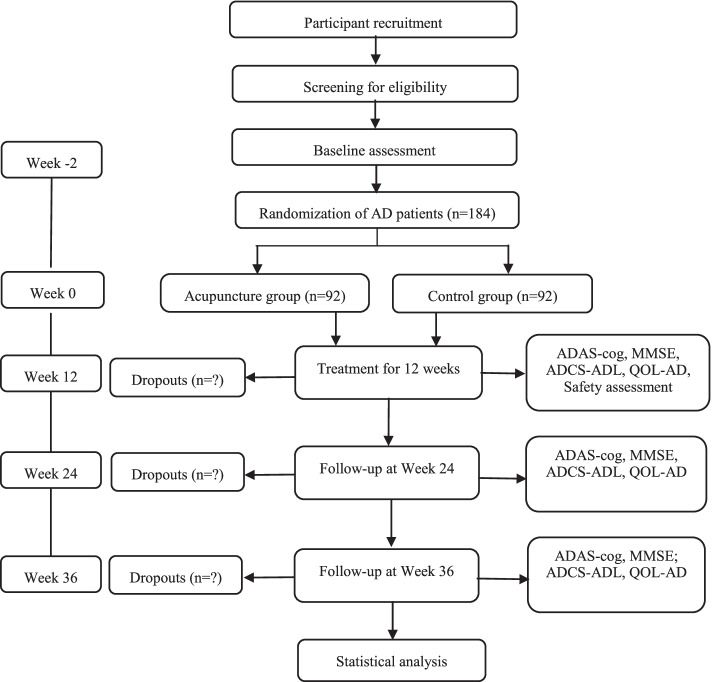
Flowchart of the trial: the participants will be randomly assigned to the acupuncture group (AG) or control group (CG) at a ratio of 1:1; all the patients will be treated for 12 weeks and be assessed at week 12, week 24, and week 36

This multicenter randomized controlled trial will be conducted from 1 February 2019 to 30 June 2022 in three hospitals. After preliminary screening, the participants will be randomly assigned to the acupuncture group (AG) or control group (CG) at a ratio of 1:1. This protocol was formulated in strict accordance with the SPIRIT reporting guidelines [39]. This trial registered identifier is NCT03810794 (www.ClinicalTrials.gov). The trial will strictly implement the principles of the “Declaration of Helsinki” (Edinburgh 2000). A flowchart of the trial is shown in Fig. 1.

## Methods/design

### Study setting

This trial will be conducted in three hospitals in Shanghai (Longhua Hospital Shanghai University of Traditional Chinese Medicine, Mental Health Center Shanghai Jiao Tong University School of Medicine, and Huashan Hospital Fudan University).

### Inclusion criteria

This study will include patients (1) aged 50–85 years; (2) who meet the AD diagnostic criteria of the National Institute of Neurological and Communicative Disorders and Stroke (NINCDS), and the Alzheimer Disease and Related Disorders Association [40]; (3) with cognitive impairment assessed using the MMSE (Chinese version), with scores ranging from 11–22 for those with primary school education and junior high school education or 11–26 for those with higher education; (4) having a Medial Temporal Lobe Atrophy Rating Scale score of ≥ 2 and ≥ 3 for patients aged < 75 and ≥ 75 years, respectively; and (5) with informed consent signed by them or their guardian.

### Exclusion criteria

Patients were excluded if (1) their cognitive dysfunction was caused by other conditions (including vascular dementia, Lewy body dementia, hormonal or metabolic abnormalities, hypothyroidism, VB12 or folic acid deficiency, delirium, or other affective and mental disorders); (2) they had severe chronic diseases, including heart disease, liver, and kidney disease, and hematopoietic system disease; (3) they were unable to cooperate with the study examinations due to aphasia, confusion, or other reasons; (4) they were taking anticoagulant drugs, including warfarin or heparin; and (5) if they had undergone acupuncture treatment within the previous 2 weeks.

### Termination criteria

In the process of clinical research, if the subject has a serious adverse event or adverse reaction, the trial will be suspended if the subject and researcher have discussed the situation and together agree that the subject cannot continue. In addition, if during the clinical research process, the subject felt that they lacked efficacy they would be given the option of suspending the trial.

### Drop out criteria

The following shedding standards were used to withdraw patients from the study. (1) Subjects who received other treatments of AD drugs during the study period; (2) subjects who did not come to the hospital for follow-up visits on time, and could not be contacted by telephone or SMS; and (3) subjects unwilling to continue to participate in the study, and who voluntarily withdraw their informed consent.

### Interventions

This RCT will recruit 184 patients with mild-to-moderate AD and randomly allocate them to two groups: AG and CG. The AG (*n* = 92) will be treated using acupuncture combined with donepezil hydrochloride 5 mg/day for 12 weeks, while the CG (*n* = 92) will only receive donepezil hydrochloride 5 mg/day for 12 weeks. The two groups will undergo routine clinical treatment throughout the follow-up period. The evaluation time points will be the 12th week, 24th week (follow-up), and 36th week (follow-up) after treatment begins.

#### Acupuncture treatment

The main acupuncture points will be GV 20, GV 24, EX-HN 1, EX-HN 3, GB12, GB39, and KI6 (acupoint location; see Table 1). Doctors will be instructed to select the combined acupoints based on traditional Chinese medicine (TCM) syndromes which are based on the TCM syndrome differentiation and diagnosis standard term reference literature [41]. Heart qi deficiency includes forgetfulness (required), indifferent expression, slow response, incomunicative, sleepy, low voice, easy to startle (with 3 or more of the above), pale complexion, and weak pulse. Kidney essence deficiency includes forgetfulness (required), sluggishness, wrong operation, loss of fluency, head tilt and back bending, and fecal and urine incontinence (three or more of the above), the pulse is thin and weak.

**Table 1:** Acupoints selected in the study

Acupoints	Location
Shenting (GV 24)	On the head, 0.5 B-cun superior to the anterior hairline, on the anterior median line.
Yintang (EX-HN 3)	At the midpoint of the line joining the medial ends of both eyebrows
Baihui (GV 20)	On the head, 5 B-cun superior to the anterior hairline, on the anterior median line.
Sishencong (EX-HN1)	On the top of the head, 1cun before and after GV20 on both sides with a total of 4 points
Wangu (GB12)	In the anterior region of the neck, in the depression posteroinferior to the mastoid process.
Zhaohai (KI6)	On the medial aspect of the foot, 1 B-cun inferior to the prominence of the medial malleolus, in the depression inferior to the medial malleolus.
Xuanzhong (GB39)	On the fibular aspect of the leg, anterior to the fibula, 3 B-cun proximal to the prominence of the lateral malleolus.
Shenmen (HT7)	On the anteromedial aspect of the wrist, radial to the flexor carpi ulnaris tendon, on the palmar wrist crease.
Neiguan (PC6)	On the anterior aspect of the forearm, between the tendons of the palmaris longus and the flexor carpi radialis, 2 B-cun proximal to the palmar wrist crease.
Taixi (KI3)	On the posteromedial aspect of the ankle, in the depression between the prominence of the medial malleolus and the calcaneal tendon.
Shenshu (BL23)	In the lumbar region, at the same level as the inferior border of the spinous process of the second lumbar vertebra (L2), 1.5 B-cun lateral to the posterior median line.

The combined acupoints of HT7 and PC6 will be applied to patients with syndromes of heart qi deficiency, while KI3 and BL23 will be applied to patients with kidney essence deficiency syndrome.

The patient is in the supine position. After disinfection, disposable stainless steel needles (0.25mm×40mm) will be used to pierce the scalp or skin surface rapidly at a 15° angle on the head or 90° on the limbs and trunk. After the needle is inserted to the prescribed depth, the needle will be twisted for about 30s for a feeling of De Qi, such as sourness, numbness and heaviness. The patients will receive treatment 30 min per session, thrice per week (every other day) for 12 weeks. Meanwhile, electro-stimulation will be applied for GV20 and GV24 acupoints (2/50 Hz, dilatational wave). All treatment will be carried out by a well-skilled senior acupuncturist.

After each acupuncture treatment, the doctor will carefully observe whether the patient has pain, subcutaneous hematoma, and other discomfort. After the treatment, the patient will be observed in the clinic for 15 min.

#### Medicine treatment

Patients will receive donepezil hydrochloride 5 mg daily for 12 weeks. During the trial, participants will be prohibited from using other medicines for AD. The test drug was allocated based on the random coding sequence of the test drug and the number of cases. Each research unit will have a trial medication administrator. The investigator will screen qualified participants. After informed consent and writing of the study medical record, the trial medication administrator will arrange participants based on the order of patient visits and drug coding from small to large distributed medicines; further, they will be registered in the “Clinical Trial Drug Use Record Form.”

#### Follow-up

All participants will be treated for 12 weeks. Subsequently, they will enter a 24-week follow-up period. In the 24th and 36th weeks, the patients will be reassessed using the MMSE, Alzheimer’s Disease Cooperative Study-Activities of Daily Living (ADCS-ADL) scale, and Quality of Life-Alzheimer’s Disease (QOL-AD) scale.

### Outcome measures

Efficacy scores are performed by clinical neurologists and psychiatrists, who are trained by Shanghai Mental Health Center. Each time the scoring is performed by two scorers separately, if their results are inconsistent, the third scorer will make a ruling. All raters do not know the group of patients at the time of scoring. Table 2 shows the intervention and assessment time points to be used during the study period.

**Table 2:**
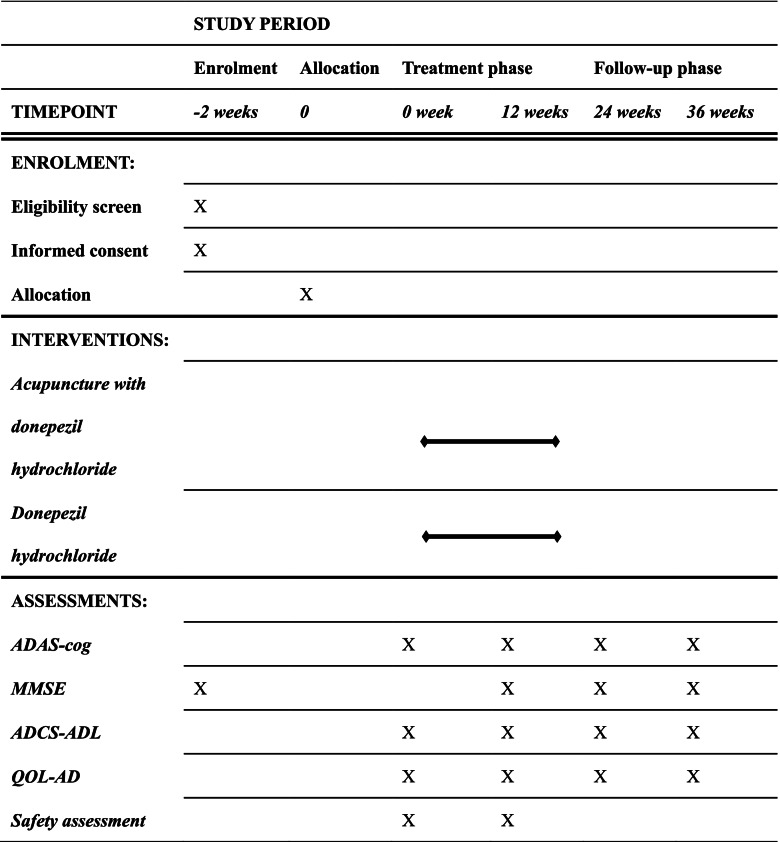
Outcome measurements at each time-point

*Abbreviations*: *ADAS-cog* Alzheimer’s Disease Assessment Scale-Cognitive Subscale, *MMSE* Mini-Mental State Examination, *ADCS-ADL* Alzheimer’s Disease Cooperative Study-Activities of Daily Living, *QOL-AD* Quality of Life-Alzheimer’s Disease

#### Basic characteristic variables

The information questionnaire will be used to collect information such as age, gender, educational background, marital status, previous history, disease course, and MMSE scores. Furthermore, participants will undergo pre-intervention blood tests to test for amyloid precursor protein, apolipoprotein E4, and presenilin-1, as well as magnetic resonance imaging (MRI) scans for definitive diagnoses. A nurse will measure and record the patient’s vital signs.

#### Primary outcome measure

The ADAS-cog evaluated at week 12 (the end of intervention) is the primary outcome of the study. The scale contains 40 items and is used to assess the severity of cognitive-behavioral impairment in patients with AD [42]. In this trial, we choose the Chinese version of the ADAS-cog scale, which contains 12 items [43, 44]. The total score of the scale ranges from 0 (no error or damage) to 75 (severe damage). A higher score indicates a more severe cognitive impairment. Generally, individuals without AD or other dementia types have a score of 5.

#### Secondary outcome measures

##### ADAS-cog at follow-ups

Participants’ cognitive function will also be assessed using the ADAS-cog scale at week 24 (follow-up) and week 36 (follow-up).

##### ADCS-ADL

The ADCS-ADL scale is mainly designed to evaluate activities of daily living in patients with dementia. Currently, the most commonly used ADCS-ADL was developed by Lawton and Brody in 1969, which is divided into physical self-maintenance and instrumental activities of daily living scales. It contains 19 domains related to making phone calls, shopping, preparing meals, doing housework, washing, using transportation, taking medicine, and taking care of money [45]. The maximum ADCS-ADL score is 54. The higher the score, the better the patient’s quality of life related to cognition. Participants will be assessed using the ADCS-ADL at baseline, week 12(the end of intervention), week 24 (follow-up), and week 36 (follow-up).

##### MMSE

The MMSE was designed in 1975 as a screening tool for patients with dementia and psychiatric disorders. It is comprised of two parts: the first evaluates direction, memory, and attention, while the second part assesses naming capabilities, execution of oral and written commands, spontaneously writing sentences, and copying complex polygons. The items are rated on correctness or error (0, wrong; 1, right) with the outcome being the total number of correct responses. The maximum MMSE score is 30 and the test is not timed [46]. MMSE scoring standards are associated with education level. A score ranging from 27 to 30 is considered normal. The MMSE will be measured at baseline, week 12(the end of intervention), week 24 (follow-up), and week 36 (follow-up).

##### QOL-AD

The QOL-AD scale was developed in 1999 to evaluate the quality of life in patients with AD and their caregivers [47]. It is comprised of 13 items regarding physical and mental health, quality of life, social, and financial assessment. The response options are divided into four levels (1, poor; 4, excellent). The score ranges from 13 to 52 [48], with 52 indicating the highest quality of life. The QOL-AD will be measured at baseline, week 12 (the end of intervention), week 24 (follow-up), and week 36 (follow-up).

### Safety

The participants will be required to undergo the following necessary clinical laboratory tests for excluding any serious disease: blood routine, urine routine, and hepatic and renal functions during the screening stage and post-treatment (week 12). The results of the tests will be recorded to evaluate the safety of this trial. Treatment-related adverse events, including local hematoma, fainting, nausea, dizziness, insomnia, vomiting, or diarrhea, will be recorded in the CRF, including the time point, severity, measures taken, causal relationship with acupuncture treatment, and final outcome. Severe adverse events will be promptly reported to the principal investigator.

### Sample size calculation

Our previous review of clinical studies on the efficacy of acupuncture with donepezil in the treatment of Alzheimer’s disease control method with ADAS-cog as a primary outcome. The sample size was determined using the results of our previous study. The main evaluation index is the change in the ADAcog score from baselinee [49] found that there is no RCT study using a similar intervention arm and to the end of 12 treatment weeks. Our previous study reported that the ADAS-cog score of the control (donepezil hydrochloride tablets) was decreased by 2.32±2.02 and the treatment groups (acupuncture combination) increased by 4.04±4.23, respectively. Based on a two-sided 5% significance level and 90% efficacy, as well as an analysis of the aforementioned data (μ1 =2.32, μ2 = 2.02, δ1=4.04, δ2= 4.23, 1-β = 0.9) using the NCSS-PASS V11.0.7 (https://www.ncss.com/software/pass/), it is found that approximately 80 participants are required in each group. Considering a dropout rate of 15% to minimize bias, a minimum of 184 participants is required.

### Recruitment

Participants will be recruited from three hospitals in Shanghai using the internet and health newspapers. Scientific lectures about the prevention and treatment of Alzheimer’s disease will be held in community service centers. Further, participants will be invited to a fixed location to have face-to-face meetings with the research liaison for assessment with respect to the eligibility criteria. Patients who have not undergone MRI within three months will be re-examined. After receiving informed consent and writing of the study medical record, the patients will undergo baseline evaluation with venipuncture. The decision to include a patient will be based on the evaluation and inspection results. This study will include 184 patients.

### Allocation

#### Sequence generation

The central randomization method will be used. The random number is uniformly issued by a dedicated person from the Data Management Center of Longhua Hospital through the statistical software SPSS (IBM SPSS statistics version 22.0, USA) network. This can ensure the random allocation being concealed.

#### Allocation concealment mechanism and implementation

The dispensing sequence is sealed in a light-tight envelope with a unique identification number. The envelope will be sent to a dedicated investigator who will write who and when opened the envelope. This identification number will appear on all reporting forms as the participant’s code. During the research process, sequence generation and allocation, recruitment, TCM syndrome differentiation, acupuncture treatment, efficacy evaluation, data management, and statistical analysis will be independently performed by different research specialists.

### Blinding

In this study, due to the nature of the intervention, the doctors and acupuncturists will not be blinded due to the assignment of the group to the operation. The data evaluation will be conducted by two evaluators who did not know the grouping. Data collection and statistical analysis are performed by a third party. Therefore, except for the differences in treatment between the two groups, all participants will be treated as equally as possible. During the intervention, the acupuncturist's treatment and the assessment of the scale of the assessor will be carried out at different times to ensure that they cannot communicate with each other.

### Data collection and management

All data will be corrected by the assessor on Case Report Form (CRF), then be a double entry into the electronic management system by two dedicated persons. The evaluator and the data entry person sign a confidentiality agreement before the study, and the data cannot be leaked. The study sponsor should retain the CRF for 5 years after the end of the study.

#### Ethics approval and consent to participate

The study protocol and research consent were approved by the Longhua Hospital Ethics Review Committee (2018LCSY060), Huashan Hospital Ethics Review Committee (2018-434), and Mental Health Center Shanghai Ethics Review Committee (2018-72).

Before participating in any research-related interventions, each participant will provide written informed consent. Before signing the consent form, the participants will be informed of all research procedures, benefits, and risks. Moreover, the patients will be informed that participation in the project is voluntary and that they can withdraw at any time. The data collected by the patient before shedding will be included in the intentional analysis. Data obtained from participants who withdraw from the study will be included in the analysis for determining the outcome. None of the participants will receive the intervention before signing the informed consent.

#### Quality control

Prior to contributing to this study, all investigators are required to attend and pass training sessions, particularly, for assessing scales and disease diagnosis (by Shanghai Alzheimer’s Disease and Related Disorders Center) assessments will be performed by individuals blinded to the group allocation. Syndrome differentiation is done by an independent TCM physician in each hospital. The syndrome differentiation and acupuncture program training was provided by Longhua Hospital, Shanghai University of Traditional Chinese Medicine.

### Oversight and monitoring

#### Data monitoring

Electronic data management system was developed by the Institute of Basic Research of the Chinese Academy of Chinese Medical Sciences to collect and manage the data.

The Shanghai Municipal Science and Technology Commission will establish an expert group which independent from researchers and conduct annual assessments of the project. The project team establishes a 3-level supervision committee to regularly supervise the outcome assessments and data management

#### Protocol amendments

The Ethics Committee of Longhua Hospital Affiliated to the Shanghai University of Traditional Chinese Medicine will conduct random checks to ensure that the study meets protocol requirements. If there is any change in the research protocol, it will be reported to the ethics committee for approval and revised in time in the clinical registration protocol.

#### Dissemination policy

The results of this study will be published in a peer-reviewed journal. Participants can request a summary of the results.

#### Patient and public involvement statement

Patient and public are not involved in the trial design and conduct. There is also no plan for patients to take part in the results reporting or dissemination.

### Statistical methods

All statistical analyses will be performed using SPSS Statistics for Windows V21.0 (IBM SPSS). Three sets of data will be used:

1. Full analysis test (FAS). This will include all randomized cases that underwent assessments at least once for efficacy analysis. Missing data, which may affect analysis, will be supplemented using the last observation data. FAS will be the main population for efficacy evaluation.
2. The protocol set (PPS, per-protocol set). This will include patients that meet the inclusion criteria specified in the trial protocol, complete all observation period plans, and do not use other drugs or treatment measures that may affect efficacy evaluation during the trial period. Efficacy evaluation will be conducted using the FAS and PPS. PPS is a secondary population for efficacy evaluation.
3. Safety analysis data set (SS, safety set). This will include all cases that use the study drug at least once and undergo post-treatment safety evaluation after randomization. The SS will be the main population for safety evaluation.

All statistical tests will be performed as two-sided, and the statistical significance will be set at *p*<0.05. Quantitative indicators will be described using the mean and standard deviation, maximum value, minimum value, and median; moreover, the classification indicators will be described using the number and percentage. For counting data, two-sample tests (including the Cochran-Mantel-Haenszel test) or Fisher’s exact probability method will be used for between-group comparisons; moreover, measurement data will be expressed as mean and standard deviation. One-way analysis of variance will be used for among-group comparisons; additionally, the least significant difference method (LSD-t test) will be used for between-group comparisons.

Demographics (age, sex, duration of illness, education level) and MMSE scores will be compared between the two groups as baseline assessments. The main time point for efficacy evaluation is 12 treatment weeks (for PPS), as well as when the group is discontinued (for the FAS population). The *t*-test will be used for between-group comparisons of other measured efficacy indicators, including analyzing the change from the baseline. The ADAS-cog, MMSE, ADCS-ADL, and QOL-AD will be analyzed using the covariance analysis model for between-group comparisons with the baseline.

## Discussion

This study aims to provide evidence regarding acupuncture application in patients with AD. In addition to the solidified study program (no syndrome/meridian differentiation), the acupoints are selected through syndrome differentiation of heart qi deficiency or kidney essence deficiency. All the involved acupuncturists are experienced, trained, and examined by the acupuncture department of Longhua Hospital for efficacy consistency.

Previous randomized controlled studies comparing electro-acupuncture and donepezil hydrochloride have shown that electro-acupuncture can improve the cognitive dysfunction of AD patients [27, 50]. We hope that through this experiment, we can explore the application of acupuncture combined with donepezil hydrochloride therapy in the conventional treatment of AD. Thus, the choice of the control group was the patients who only take donepezil hydrochloride, which meets the requirements of clinical guidelines.

This study will observe the treatment efficacy after 12 weeks; however, AD is a chronic disease requiring long-term management. Other than contributing to the literature, the results should be translated into community-based public health plans that benefit the population. By cooperating with various elderly activity centers and voluntary welfare organizations, we will extend our program to the community, observe the clinical efficacy of long-term acupuncture, and apply evidence-based interventions to the community.

Compared with other clinical research protocols for acupuncture treatment for AD, our protocol will be the first study protocol concerning the effect of acupuncture based on syndrome differentiation for AD. Study [51] has shown that the orderly pattern evolution defined by Chinese medicine was starting from kidney deficiency. The cascade hypothesis of Kidney deficiency in AD and its sequential therapy based on Kidney-reinforcing was proposed. Therefore, our treatment protocol includes some acupoints for the treatment of Kidney deficiency syndrome of AD, such as Zhaohai (KI-6), which reflects the advantages of TCM syndrome differentiation.

## Study status

Currently, we are recruiting participants for this trial. The first patient was enrolled in Feb 2019, and the study is expected to end in June 2022.

## Data Availability

Not applicable.

## Abbreviations

AD: Alzheimer’s disease
ADCS-ADL: Alzheimer’s Disease Cooperative Study-Activities of Daily Living
ADAS-cog: Alzheimer’s Disease Assessment Scale-Cognitive Subscale
AG: Acupuncture group
CG: Control group
FAS: Full analysis test
MMSE: Mini-Mental State Examination
QOL-AD: Quality of Life-Alzheimer’s Disease
TCM: Traditional Chinese Medicine
